# A retrospective study exploring how South African newspapers framed Schizophrenia and other psychotic disorders over an 11-year period (2004–2014)

**DOI:** 10.1186/s12888-022-04276-5

**Published:** 2022-10-28

**Authors:** Nombuso Masinga, Patrick Nyamaruze, Olagoke Akintola

**Affiliations:** 1 Discipline of Psychology, School of Applied Human Sciences, University of KwaZulu-Natal, Durban, South Africa; 2 School of Public Health, Faculty of Community and Health Sciences, University of the Western Cape, Cape Town, South Africa

**Keywords:** Non-communicable diseases, Media framing, Mental health care, Schizophrenia, Newspaper, South Africa

## Abstract

**Background:**

The way schizophrenia is portrayed in the media contributes to the dissemination of misinformation about the symptoms, causes, and treatment of mental disorders and has the potential to perpetuate or mitigate the stigmatization of schizophrenia. While research on the news media’s role in exacerbating or mitigating the stigmatization of schizophrenia has been conducted widely in other contexts, our search did not yield any study on media framing of schizophrenia in South Africa. Therefore, this study used the framing theory to examine the media framing of schizophrenia following the enactment of two mental health policies in South Africa.

**Methods:**

We examined 216 news stories that covered the schizophrenia spectrum and other psychotic disorders from 20 South African newspapers retrieved from the SABINET – SA Media online archive over an 11-year period (2004–2014). Thematic analysis was used to analyse the news stories.

**Results:**

The findings show that most of the news stories had problems as their main frame. These were followed by stories framed to diagnose the causes of schizophrenia and other psychotic disorders; and stories that made moral judgements about issues around the schizophrenia spectrum and other psychotic disorders. Stories that were classified as suggesting remedies were relatively less frequent. A common thread in the news stories was the misperceptions about schizophrenia and other psychotic disorders. Media framing of the cultural interpretations of schizophrenia and other psychotic disorders tended to be derogatory and therefore stigmatising. Most news stories framed schizophrenia and other psychotic disorders as mainly caused by using psychoactive drugs/substance with Cannabis as the most frequently mentioned psychoactive drug.

**Conclusion:**

The study underscores the role of media analyses in framing schizophrenia and other psychotic disorders following the development of major mental health policies. The study showed that the media framing of schizophrenia could perpetuate stigmatisation, discrimination and social rejection of people with lived experiences of the condition. Our findings highlight the need for collaboration between researchers and the media to enhance opportunities for improved and more nuanced reporting of mental health issues.

## Introduction

Schizophrenia affects approximately 24 million people worldwide [1] and it is one of the top 20 leading contributors to years lost to disability globally [2]. In the DSM-5, schizophrenia is classified under the Schizophrenia Spectrum and Other Psychotic Disorders category. Schizophrenia is a complex disorder with a range of symptoms including cognitive, behavioural, and emotional dysfunctions, but no single symptom is pathognomonic of the disorder [3].

Schizophrenia and other psychotic disorders are characterized by significant impairments in reality testing and alterations in behaviour manifest in positive and negative symptoms [4]. Examples of positive symptoms are persistent delusions, persistent hallucinations, disorganized thinking (that typically manifests as disorganised speech), grossly disorganised behaviour and experiences of passivity and control [4, 5]. Negative symptoms include avolition, blunted or flat affect and psychomotor disturbances [4, 5]. Positive symptoms tend to diminish naturally over time, whereas negative symptoms often persist and are closely associated with a poor prognosis [4]. In addition to the positive and negative symptoms, people living with schizophrenia experience multiple persistent symptoms and are typically accompanied by impairment in cognitive functioning and other psychosocial problems [4, 5].

While a mix of psychosocial interventions and medications are available for the treatment of the symptoms of schizophrenia [5], only a small number of people living with schizophrenia recover completely [3]. Current treatments (antipsychotic medications) assist in alleviating some of the positive symptoms experienced by individuals living with schizophrenia. However, people with lived experience of schizophrenia often continue to experience residual symptoms and in some medicated patients, residual hallucinations and delusions do not completely resolve [6, 7]. Psychosocial treatments, in particular cognitive behavioural therapy, is also used for the treatment of schizophrenia and offer additional promise as an effective treatment for those positive symptoms that are persistent or medication resistant [7].

With adequate treatment and support, most individuals with schizophrenia are able to live a meaningful and satisfying life, in the presence or absence of symptoms [8]. However, structural and psychosocial factors such as lack of mental health services and low perceived treatment need may compromise treatment outcomes for individuals diagnosed with schizophrenia [9]. People with schizophrenia are less likely to seek care than the general population owing to ignorance and stigma surrounding the condition [10, 11].

While there have been significant advances in treatment globally, in past decades, the stigmatization and discrimination of individuals with a mental health condition has persisted [12, 13]. This is particularly true for South Africa where one study found that mental health stigma perpetuated by family members, peers, community members and health care providers was rife often leading to delays in help-seeking among patients [13].

Entman’s [14] framing theory provides a useful lens for exploring media representations of schizophrenia, as well as how the media shapes public perceptions and policies around schizophrenia. Entman [14] defines framing as the process of assembling a narrative that highlights the connections among a few elements of perceived reality (that have been culled) to promote a particular interpretation. Entman [14] proposes four main purposes of frame analysis which encompass the core business of strategic framing: (1) to define problems, (2) to diagnose causes, (3) to make moral judgments, and (4) to suggest remedies.

Perhaps most concerning of all, the media often creates a negative imagery of mental illness that emphasises dangerousness, criminality and unpredictability [15]. The media can also trivialize mental illness, either by promoting mental illness as not being severe or being less severe than it really is [16]. A media analysis conducted in the Czech Republic and Slovakia found that a vast majority of the analysed articles presented individuals living with a mental health condition as perpetrators of aggressive behaviour [17]. The way schizophrenia is framed in the media could contribute to the dissemination of misinformation about the symptoms, causes, and treatment of schizophrenia as well as other forms of severe mental illness.

In South Africa, the Mental Health Care Act, No. 17 of 2002, and the Mental Health Care Regulations of 2003 were developed to, among other provisions, guide the provision of treatment and care to people living with mental health conditions [18, 19]. Among others, the policies have provisions that seek to protect the human dignity and privacy of people living with mental health conditions [18, 19]. One would expect that the enactment of these policies would positively influence the treatment and care as well as stigma and discrimination experienced by people living with a mental health condition. An analysis of print media in the years following the enactment of these policies could provide insight into how the media framed issues relating to the treatment and care of people living with schizophrenia and other psychotic disorders as well as stigma and discrimination experienced by this population in South Africa. However, we know very little about how the media framed issues relating to schizophrenia and other psychotic disorders during this period; our literature search did not yield any media study. Therefore, this study used the framing theory to explore how the South African print media framed schizophrenia and other psychotic disorders by examining newspapers published in the 11-year period following the implementation of these policies. The findings of the study could help inform mental health policies and enhance our understanding of how to engage the media in South Africa.

## Methods

We used SA Media – located on the SABINET search engine to search for news stories relating to schizophrenia spectrum and other psychotic disorders. SA Media is a database that provides full-text electronic records (from 1978 onwards) of South African print media. SA Media contains functions for identifying news stories with various terms/key words. The searches were conducted between May and June 2015.

### Newspaper search and selection strategy

We retrieved newspapers available in SA Media using a search strategy that the research team jointly developed. We developed a set of inclusion criteria through an iterative process to assist in retrieving newspapers from SA Media. The criteria for inclusion were that newspapers had to: (1) be classified as a newspaper published in South Africa; (2) be published in English; (3) be published for a fairly broad readership (e.g., newspapers that were printed solely for the police forum or financial mail were excluded as we felt they were not widely read by the general public); (4) the news story had a term related to schizophrenia and other psychotic disorders in the heading or the full text of the news story. Twenty newspapers met the inclusion criteria. The inclusion criteria were set to include newspapers published for a 11-year period (from 1 to 2004 to 31 December 2014).

### Selection strategy

Using the inclusion criteria described earlier, the first author (NM) searched for news stories from the twenty newspapers on the online database, SA Media, using specific search terms. All the terms that are related to schizophrenia, generally used in the literature as well as the DSM-5, were used as search terms instead of restricting the search to only the terms “the schizophrenia spectrum and psychotic disorders”. This was to ensure that as many news stories as possible were retrieved. These search terms are “schizophrenia”; “psychosis” (and its variant spellings); “psychotic disorders”; “schizotypal”; “schizoaffective”; “schizophreniform”; and “catatonia”. We conducted the search of these terms in the titles as well as the full texts of the news stories. Therefore, if the title or the full text of the news story had at least one of the exact search terms, those news stories were retrieved from SA Media.

### Selection of news stories

**Fig. 1:**
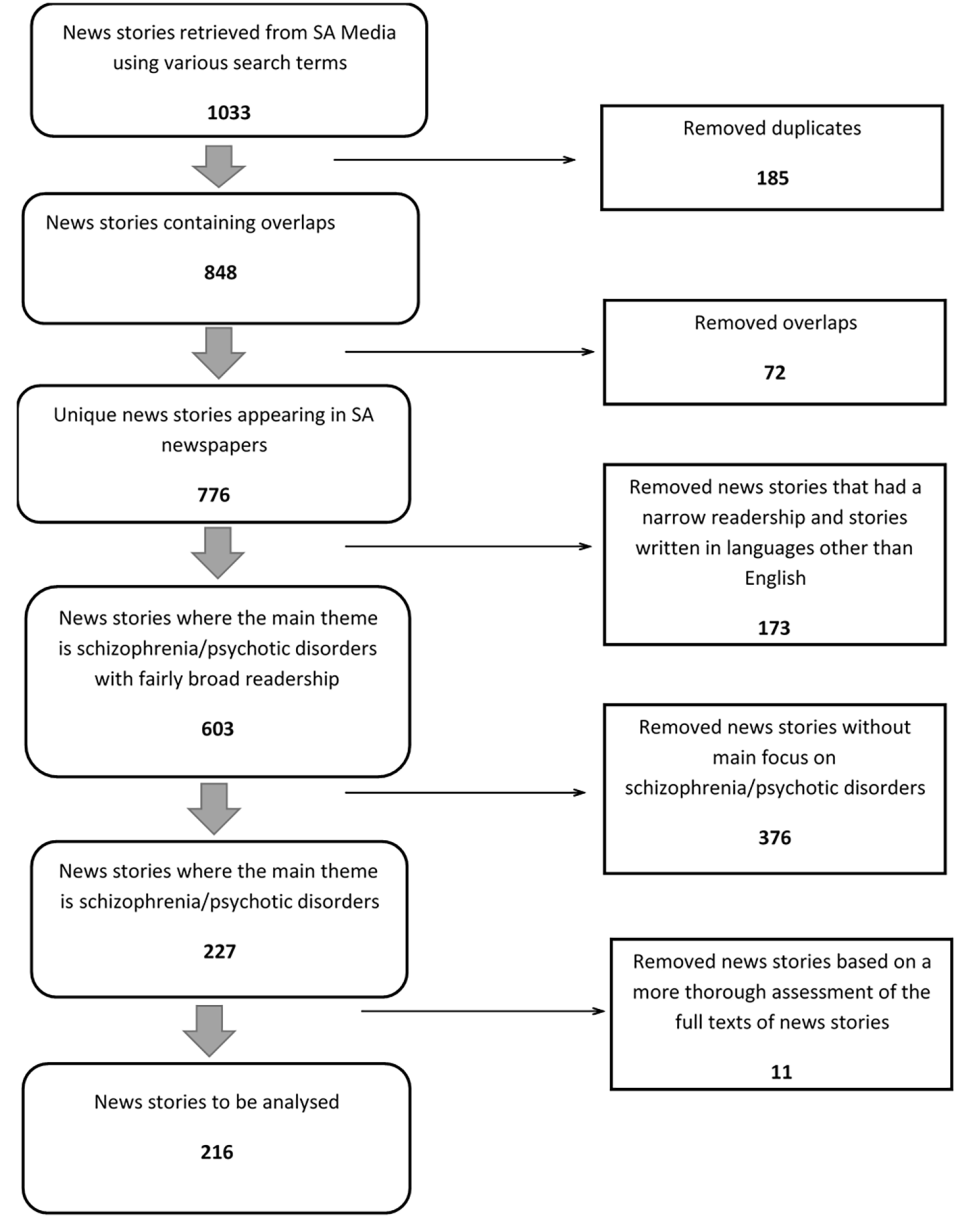
Flow chart depicting selection of news stories

A total of 1033 news stories were retrieved from the selected 20 South African newspapers and 216 were selected for analysis as shown in Fig. 1.

### Analysis of news stories

We employed Braun and Clarke’s [20] thematic analysis as our methodological orientation for the study and used it to conduct an analysis of the 216 news stories that made up the sample. The first (NM) and second (PN) authors worked collaboratively for analysis of these news stories to validate the data and regularly consulted with the third author (OA). Various themes were developed regarding the way in which South African print media frames the schizophrenia spectrum and other psychotic disorders. We used both the deductive approach and the data-driven inductive approach [21]. This approach complemented the research questions and theoretical approach by allowing the tenets of the framing theory to be integral to the process of deductive thematic analysis while allowing for themes to emerge directly from the data using inductive coding. An inductive approach meant that the identified themes were strongly linked to the data themselves [20].

We conducted inductive thematic analysis following the six steps outlined by Braun and Clarke [20]. Once the sample news stories had been selected, NM, in the first step, familiarised herself with these news stories by reading and re-reading the stories whilst making notes before coding began. NM was fully cognisant of Entman’s [14] description of frames which would later (in the second step) form the basis for organising the data into broad meaningful concepts.

These concepts which constituted *a priori* main themes are: (i) problems, (ii) causes, (iii) moral judgements, and (iv) suggested remedies. In the second step, NM organised the data under these four *a priori* themes by generating an initial list of codes relating to dominant frames related to these four themes in each news story. Frames related to the costs and benefits of the causes of schizophrenia were classified under the problems theme. Frames that identify the forces creating the problem were classified under the causes theme [14]. Frames that describe judgements made about causal agents related to schizophrenia and their effects were classified under the moral judgements theme and frames that describe treatments for the problems and predictions of their likely effects were classified under the theme called suggested remedies [14]. The initial coding was then reviewed and modified by OA. Thirdly, the codes were organised into potential sub-themes under each of the four concepts/main themes and the data relevant to each sub-theme was collated. In the fourth step, in order to determine if the sub-themes were relevant in relation to the whole data, PN conducted a general review of the themes and sub-themes, and these were reworked where they proved problematic, for instance, if there was not enough data. Fifth, the sub-themes were defined and named, following revision. In the last and final step, we present a report on the final analysis of the news stories. All the authors revised, finalized and agreed on the themes and sub-themes.

## Findings

### Characteristics of data set

The 20 newspapers that met the inclusion criteria for analysis are shown in Table 1 while the characteristics of the 216 news stories that constituted our sample are shown in Table 2.

**Table 1:** Newspaper characteristics covered in analysis

Geographical circulation of newspapers	Newspapers	Frequency	Publisher	Circulation
**National Newspapers**				
	Citizen	Mon-Fri	CTP/Caxton	54 689
	City Press	Sunday	Media24	94 290
	Mail & Guardian	Weekly	M & G Media Ltd. Johannesburg	30 286
	New Age (The)	Daily	TNA Media	Not registered with ABC
	Star (The)	Mon-Sat	Independent Newspapers, Johannesburg	85 567
	Sowetan	Daily	Avusa Media Ltd., Johannesburg	92 453
	Sunday Independent (The)	Weekly	Independent Newspapers, Johannesburg	Not registered with ABC
	Sunday Times	Weekly	Avusa Media Ltd., Johannesburg	338 532
	Sunday Tribune	Weekly	Independent Newspapers, Johannesburg	61 035
	Times (The)	Weekly	Avusa Media Ltd., Johannesburg	109 484
**Provincial Newspapers**				
Eastern Cape	Daily Dispatch	Mon-Sat	Avusa Media Ltd, Johannesburg	23 585
	Herald (The)	Mon-Fri	Avusa Media Ltd, Johannesburg	21 285
	Weekend Post	Weekly	Avusa Media Ltd, Johannesburg	18 441
Gauteng	Pretoria News	Mon-Sat	Independent Newspapers, Johannesburg	14 401
KwaZulu-Natal	Business Day	Daily	BDFM Publishers (Avusa Media Ltd)	25 753
	Daily News	Daily	Independent Newspapers, Johannesburg	25 091
	Independent on Saturday	Saturday	Independent Newspapers, Johannesburg	39 061
	The Witness	Daily	Media 24	14 879
Western Cape	Cape Argus/Argus Weekend	Mon-Sun	Independent Newspapers, Johannesburg	31 197
	Cape Times	Daily	Independent Newspapers, Johannesburg	54 689

**Table 2:** Characteristics of news stories with main focus on the schizophrenia spectrum and other psychotic disorders (N = 216)

Variable	Sub-variable	Total	%
**Newspaper source**			
**National newspapers**		**97**	**44.9**
	Citizen City Press Mail & Guardian New Age (The) Star (The) Sowetan Sunday Times Sunday Tribune Sunday Independent (The) Times (The)	10 06 12 01 39 08 05 05 05 06	4.6 2.8 5.6 0.5 18.1 3.7 2.3 2.3 2.3 2.8
Provincial newspapers		**119**	**55.1**
	***Eastern Cape newspaper news stories*** Daily Dispatch Herald (The) Weekend Post	**17** 09 03 05	**7.9** 4.2 1.4 2.3
	***Gauteng newspaper news stories*** Pretoria News	**13** 13	**6.0** 6.0
	***KwaZulu-Natal newspaper news stories*** Business Day Daily News Independent on Saturday (The) Witness (The)	**40** 10 18 02 10	**18.5** 4.6 8.3 0.9 4.6
	***Western Cape newspaper news stories*** Cape Argus/Argus Weekend Cape Times	**49** 32 17	**22.7** 14.8 7.9
Year of publication	2004 2005 2006 2007 2008 2009 2010 2011 2012 2013 2014	21 17 19 24 18 10 20 15 25 32 16	9.7 7.9 8.8 11.1 8.3 4.6 2.3 6.9 11.6 14.8 7.4
Publisher	Avusa Media Ltd. CTP/Caxton Media24 M&G Media Ltd. Independent Newspapers TNA Media	46 10 16 12 128 01	21.3 4.6 7.4 5.6 59.3 0.6

### Framing

The dominant frames which were the most salient aspects of each news story were identified. Following Entman’s [14] conceptualization of framing, four dominant frames were drawn which were: “problems”, causes”, “moral judgements”, and “suggested remedies”. Table 3 presents the sub-themes that were identified under each of the main themes.

**Table 3:** Print media frames of the schizophrenia spectrum and other psychotic disorders

Problems	Causes	Make moral judgements	Suggest remedies
Included stories discussed: (a) public perceptions/stigma/general misconceptions about illness (b) incidence/prevalence (c) social implications of illness on people with a mental health condition (d) shortage of mental health professionals (e) adherence to medication and beliefs about illness/treatment (f) lack of funding for mental health initiatives (g) NGOs and communities taking action	Included stories discussed: (a) effects of drugs/substances on mental health (b) new, on-going, or proposed research on causes (c) evidence of cause or cure	Included stories discussed: (a) call for action (b) opposition of particular solution	Included stories discussed: (a) proposed solution (b) implemented solutions

There were 171 (79.0%) news stories that had problems related to schizophrenia as their dominant frame, and 93 (43.1%) news stories had the causes of schizophrenia and other psychotic disorders as their dominant frame. Twenty-one (9.9%) news stories had suggestion of remedies as their dominant frames, and 38 (17.7%) news stories had moral judgements about issues and actions relating to the schizophrenia spectrum and other psychotic disorders as their dominant frames.

**Fig. 2:**
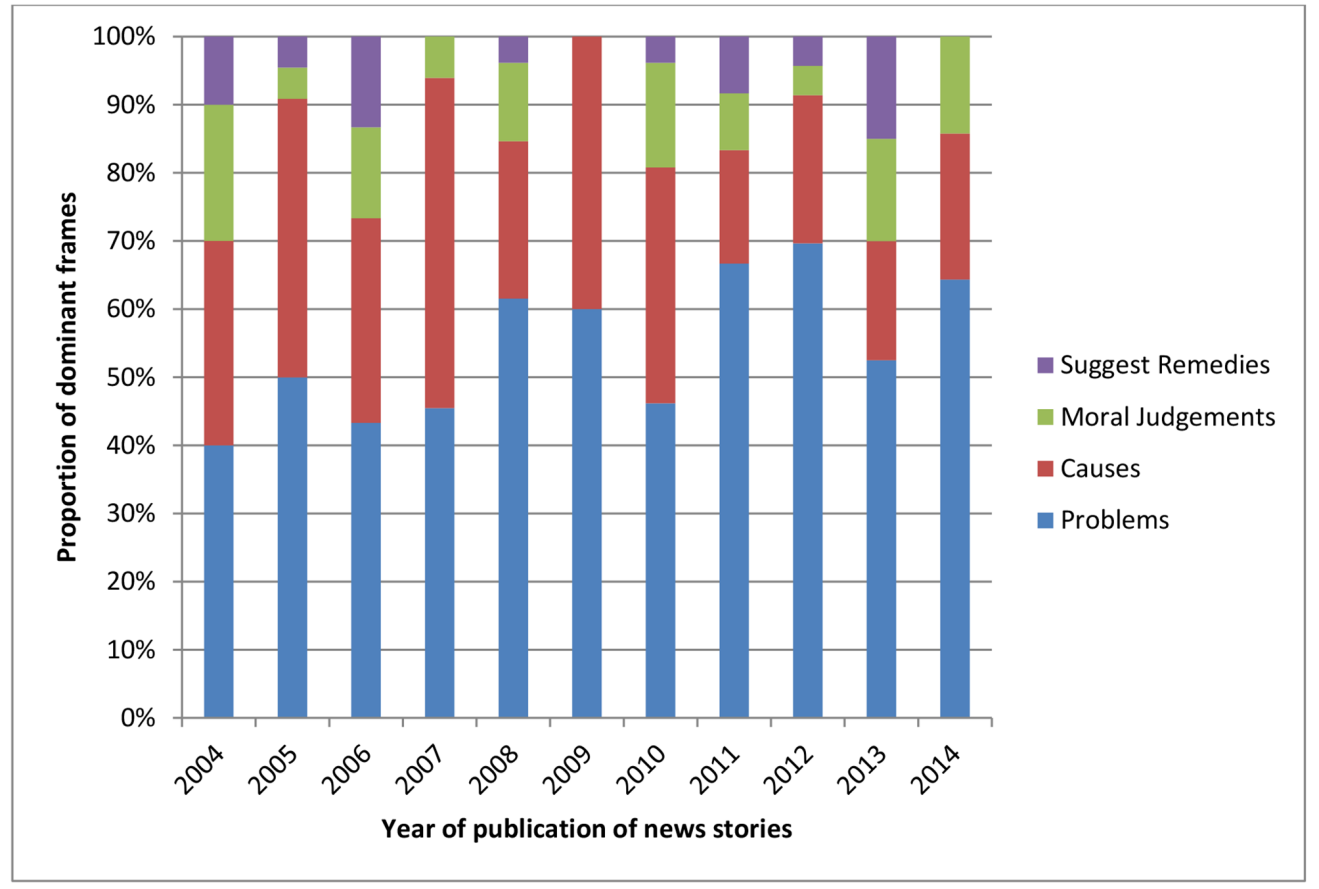
Proportion of dominant frames in each year of analysis

The dominant frames with the highest frequency each year were problems as shown in Fig. 2, while suggested remedies were the least reported each year.

## Problems

This theme encompassed the main issues regarding the schizophrenia spectrum and other psychotic disorders that frequently appeared in news stories and the context in which they were framed that suggested problem definition.

### (A) public perceptions/stigma/general misconceptions about illness

News stories illustrated actions taken by the families of individuals with a mental health condition driven by their perceptions, misconceptions and stigma. In some cases, schizophrenia was perceived as a spiritual problem that required spiritual intervention.

“Mduduzi suffers from a mental illness; his parents brought him to St John Apostolic Church in Etwatwa, East Rand seven months ago. They hope church rituals such as induced vomiting by swallowing “holy water” will rid him of his demons…Prayers and rituals cure patients better than modern medicine, says St John’s minister Lucas Kunene.” (The Star, 15 July 2004)

Other news stories reported on how culture played a role in influencing the perceptions of people regarding mental illness. Although the presence of mental illness was acknowledged in some cultures, derogatory terms were used to describe people with mental health problems:

“…naturally this does not mean that there are no people with mental illness in Zulu speaking communities. There are. We just don’t say a person is autistic, schizophrenic or suffering from manic depression. We say umuntu uyahlanya: a person is mad. We say umuntu usangene: a person is insane or, if we are feeling particularly colourful, that ngathi azithathi kahle ekhanda: a person has a brain malfunction.” (The Business Day, 24 June 2010)

A common thread in the news stories was the misconceptions and the perceptions the public had about mental illness mainly emanating from media programmes. Such misconceptions reported by the news stories led to beliefs that people living with schizophrenia should be institutionalised:

“Despite what you have learnt from poorly researched movies and TV programmes, schizophrenia does not involve having multiple personalities...but if someone with the disorder commits a crime everyone assumes that the schizophrenia is to blame…sometimes this even renews calls for all “mental” patients to be locked up “for the good of the public”.” (The Citizen, 12 June 2012)

### (B) Incidence/prevalence

Most news reports of incidence/prevalence claimed that schizophrenia was very common in South Africa and that most people who suffer from it have never been properly diagnosed. The news stories also reported on the proportion of the population that was affected by schizophrenia.

“Schizophrenia affects about 1% of the population and usually manifests in males aged 15 to 25 and females 25 to 35, according to Dr Yusuf Moosa, senior psychiatric specialist with Gauteng Community Mental Health Department.” (The Star, 10 October 2005)

“According to Wikipedia between 0.6% and 1% of the world’s population suffers from schizophrenia. That translates to somewhere between 3000 and 5000 people in South Africa.” (The Herald, 16 April 2008)

### (C) Social implications of illness on people with a mental health condition

Some news stories documented the personal experiences of people with schizophrenia, detailing how they suffer in silence. The news stories further documented the difficulties that patients encountered in their social spaces, particularly in families and in the workplace as they endeavoured to keep their diagnosis a secret:

“Due to the stigma and misunderstanding surrounding the illness, my son will not tell his employer that he has it, so the same level of performance is expected as from any other person…his father does not accept that he has schizophrenia…” (The Independent on Saturday, 08 July 2006)

### (D) shortage of mental health professionals

A commonly reported problem in the news stories was the shortage of mental health care professionals. Indeed, these new stories placed a general emphasis on South Africa’s clinician to patient ratios: The issue of shortage in mental health professionals was portrayed as pervasive.

“…the public sector faces a severe shortage of psychiatrists and psychologists, he said. Only 14% of the 2692 clinical psychologists registered with the Health Professions Council SA are working in the public sector, just 0,32 psychologists per 100 000 of the population and 0,28 psychiatrists per 100 000.” (The Cape Argus, 31 March 2014)

The shortage of mental health professionals within the mental health care systems was framed as a symptom of even bigger problems that existed within the health care system such as the inadequate number of mental health care facilities, few professionals being trained per year, as well as few professionals that worked in the public sector.

### (E) adherence to medication and beliefs about illness/treatment

Poor adherence to treatment among patients with schizophrenia was generally reported as the root cause of relapses. News stories emphasised how patients failed to completely follow their medication due to several factors which included the beliefs the patients had about medication:

“If medicated, many may be stabilized. But often, patients have to be persuaded to take daily medication. Many think it will extinguish their creative spark, Katz says.” (The Star, 15 July 2004)

Other news stories reported on the implications of patients’ poor adherence which ultimately caused relapses:

“Poor compliance is definitely the most important cause of relapses, and relapses are one of the biggest problems with the illness.” (The Cape Argus, 22 September 2005)

### (F) lack of funding for mental health initiatives

Several of the news stories illustrated the insufficient funding of mental health initiatives, whereby mental health providers had to supplement for shortfalls in their budget. One of the news stories reported the experience of a non-governmental organization providing mental health services:

“...mental health was the least funded and the least marketable…mental health is not sexy….here [in the NGO] we give them voice. There’s no stigma,“ she said…but funding is a challenge. “…we receive 36% of our funding from the DOH [Department of Health], but we have to fund raise for the remaining funds.” (The Cape Argus, 15 July 2013)

### (G) NGOs and communities taking action

News stories recognized that NGOs, churches, as well as community members were taking matters into their own hands, albeit in an inhumane manner. This was driven mainly by the misconceptions surrounding mental illness, as well as the shortages in resources (insufficient MHC facilities and MH professionals).

“Mduduzi hasn’t seen his parents for several weeks, but hopes he will go home soon. He knows that if he becomes hyperactive or aggressive, he’ll be handcuffed. But he says he feels fine and has stopped taking his medication he receives on his rare visits to a local clinic….Kekana sees the chaining of mentally disabled people, which happens at several St John’s congregations, as a last resort. “Some of the priests chain them because they get so wild they can kill people,“ he says… Rensburg says NGOs - themselves badly funded - need to function as a “deadlock” in cases where parents and church maintain they’re doing the right thing.” (The Star, 15 July 2004)

However, not all organisations treated individuals living with a mental health condition inhumanely. Other news stories reported on the active role played by certain organizations in bringing awareness in communities as they felt that government was not doing enough to address mental health-related issues:

“The organisation’s work, she said, involved bringing awareness to communities of what mental health was and how victims and families of victims should approach problems.” (The Herald, 17 October 2007)

## Causes

This theme encompassed the main issues regarding the schizophrenia spectrum and other psychotic disorders that frequently appeared in news stories and the context in which they were framed that suggested causal analysis or an interpretation of the causes.

### (A) Effects of drugs/substances on mental health

A major cause of problems reported in the sample was the use of substances and the effects it had on individuals living with a mental health condition, or how it triggered mental illness, particularly psychosis. Cannabis was commonly framed in several articles as a growing concern. A number of these news stories reported on various studies that investigated the relationship between MH and cannabis. The findings of most of these studies were that cannabis almost always exacerbated symptoms of psychosis in people who already had mental illness, and that the use of cannabis was consistently associated with psychotic symptoms which included disabling psychotic disorders. The studies reported in the news stories also found that cannabis aggravated schizophrenia. The news stories further reported that people have limited knowledge about the link between psychoactive substances and mental illness.

“People should know about this; they know you have a risk of lung cancer with smoking but nobody knows about the risk of psychosis with cannabis.…Experts believe that about 10% of schizophrenia cases are triggered by cannabis.” (The Cape Argus, 02 December 2004)

### (B) New, on-going, or proposed research on causes

Scientists and psychiatrists were mostly quoted as the ones discussing existing research on possible causes. One of the news stories discussed research that was conducted for a doctoral thesis on the causes of schizophrenia, the remaining news stories discussed research that was conducted in psychiatric institutions in various countries (South Africa, Scandinavia, Britain, France, US). Several news stories reported that most of the research focussed on Cannabis and its link to psychosis. The studies found that regular cannabis use could trigger schizophrenia in some people.

“A Dutch study found that heavy users, who smoked two or more cannabis joints a week were almost seven times more likely to have psychotic symptoms three years later. And research in France found smoking at that level can trigger psychosis in people with a family history of psychiatric illness - Daily mail.” (The Star, 13 July 2004)

Despite reporting on the link between drug use and schizophrenia, a number of news stories noted that the exact causes of schizophrenia and psychosis were not well understood even in the science field. Several news stories reported that Stellenbosch University’s medical school was at the cutting edge of new research into injectable medications for patients with schizophrenia.

“What we won the right to test is a new-generation anti-psychotic in a longer-acting injectable form. We are the first to be conducting trials worldwide using it as a first line treatment for schizophrenia…Emsley said.” (The Cape Argus, 22 September 2005)

### (C) evidence of cause

There was a consensus among the news stories that schizophrenia is a chronic mental disorder characterised by recurrent episodes of psychosis in which the individual experiences hallucinations - usually auditory - as well as delusional ideas, disorganized behaviour and illogical thought processes. Cannabis use was again consistently framed as the major trigger for schizophrenia in these news stories which referenced evidence of various research findings.

“Peter Stoker, of National Drug Prevention Alliance, said: “This research gives the lie to apologists for cannabis who have consistently tried to find something other than cannabis to blame for mental illness. It makes it clear that everyone who smokes is vulnerable.” (Daily News, 12 July 2004)

## Moral Judgements

This theme encompassed moral judgements that were reported about actions proposed and taken, opposition to particular solutions as well as support of particular solutions.

### (A) call for action

The news stories called on both the national and provincial governments to increase mental health services and resources including the number of beds in mental health facilities.

“…but Daniels said she would like to see an urgent audit of the availability of mental health beds countrywide, with the aim of providing more. We also need increased community psychosocial mental health services to assist and support people living with mental disability on their journey to recovery.” (The Saturday Argus, 27 July 2013)

One news story highlighted the benefits of early detection of mental illness and the need to prioritise issues related to mental illness.

“Making fun of people in institutions like Weskoppies and Sterkfontein [mental health institutions] and mocking those with mental illness must stop…if people are diagnosed early, many disastrous consequences can be prevented. Just like detecting cancer early can help people survive, so can people with mental illnesses who are diagnosed early enough be helped...there is a desperate need to treat the problem of mental illnesses more seriously.” (The Star, 19 May 2014)

### (B) opposition of particular solution

News stories frequently reported on the government’s policy that promoted deinstitutionalization after psychiatric treatment. This was in an effort to cut costs and reintegrate patients with psychiatric illnesses into communities. However, the policy had been reported in many cases to have backfired. Several issues were discussed by the news stories in relation to deinstitutionalization. (1) it was reported that the problem with this is that after leaving these institutions, people who suffered from schizophrenia were not automatically ready to continue with everyday life; (2) once de-institutionalised, people with schizophrenia still suffered from negative symptoms like lack of drive, low motivation and social withdrawal – which made it difficult for them to interact with friends and family, more specifically people at work; (3) families struggled to cope and many patients ended up being hospitalized partially due to the fact that they stopped taking their medication; because South Africa has underfunded institutions which gave rise to amateur efforts to provide assistance.

“Particularly attractive is the notion that care of the mentally disabled should be de institutionalised and relocated within communities. In an ideal world, this would make good sense. But our world isn’t ideal. The main effect of the policy, therefore, is underfunded institutions, a situation that gives rise to amateur efforts to provide assistance.” (The Star, 21 July 2004)

Another policy that the news stories opposed was one in relation to the 72-hour process employed by secondary institutions before a patient is transferred to a tertiary institution or sent home. The problem with this appeared to be the large numbers of patients who required long term care, that are released back into the community, which gave rise to numerous issues that were mostly discussed above.

“In another policy change aimed at keeping patients out of long-term care, hospitals are obliged to keep psychiatric patients in short-stay wards to see whether they can stabilize them rather than sending them to psychiatric hospitals.” (The Mail and Guardian, 07 September 2006)

## Suggestion of remedies

This theme encompassed proposed remedies/solutions which included policy options, solutions that have already been implemented, as well as the cancellation or removal of solutions.

### (A) proposed solution

Efforts to train and educate both mental health facility personnel and the public were reported. One of the news stories reported on promises made of increased funds and resources for mental health services:

“A [mental health] action plan is being finalised in consultation with stakeholders and…resources will be allocated to fund the priority activities, said Maja. The answer is more beds.” (The Mail and Guardian, 18 April 2013)

### (B) implemented solutions

The government’s “deinstitutionalisation” policy was reported in many news stories, and it was described as an effort of cutting costs and integrating patients in communities by discharging them back to their families. Another solution that had been implemented was the MHCA (17) of 2002.

“The mental health care act (17) of 2002 was promulgated in December 2004 to address the problems and protect the rights of mental health care users. The DOH has since embarked on extensive quality evaluation of healthcare facilities around South Africa.” (The Star, 04 July 2006)

“…unlike the previous health care system which followed a curative and hospital-based approach for mental health, the new system emphasises prevention and health promotion with mental health services integrated into the primary healthcare system.” (The Cape Argus, 11 December 2008)

There were also programmes that were reported with aims of creating awareness around mental illness with a huge focus on eradicating the stigma that was associated with it and to correct/clarify any misconceptions through educating communities as well as patients with mental illness.

“Mokgata said SAFMH had programmes to educate and empower communities about metal illness. He said one of the primary objectives was to remove the stigma associated with the condition of mental health.” (The Sowetan, 19 July 2006)

## Discussion

Our study aimed to provide insight into how the South African print media framed issues relating to schizophrenia spectrum and other psychotic disorders in the years following the enactment of two major health policies. A common thread in the news stories was the misperceptions about schizophrenia and other psychotic disorders. Most of the news stories reported that people living with schizophrenia and other psychotic disorders were perceived by society as violent and dangerous and that this was a justification for institutionalising them. Some news stories reported how people living with schizophrenia are abused and sometimes put in handcuffs. An online study by Owen [22] analysing 41 movies for depictions of schizophrenia found that the majority of characters in the movies displayed violent behaviour toward themselves or others. The framing of schizophrenia in the media may trigger and sustain the false stereotype that people with schizophrenia are prone to violence and unpredictable behaviour. Nonetheless, it was reassuring that some of the news stories in our sample had frames debunking this myth by indicating that people with schizophrenia were frequently victims of violence [23]. However, none of these news stories referenced the provisions of the Mental Health Policies of 2002 and 2003 that requires that every individual or organization providing mental health care to protect people living with a mental illness from exploitation, abuse and degrading treatment [18, 19].

Some of the frames reflected the societal perceptions of the illness as a spiritual problem which required treatment by spiritual people. A study among Xhosa people in South Africa found that schizophrenia may be attributed to the influence of ancestors or to bewitchment. This suggests the need for a better understanding of cultural frameworks in treating the illness [24]. Our study further showed how the print media framed the cultural interpretations of schizophrenia and other psychotic disorders. These portrayals tended to be derogatory and therefore stigmatising. Rhydderch and colleagues [25] conducted a media analysis of mental illness in England and found that people had negative attitudes towards people with schizophrenia and other psychotic disorders. In addition, our media analysis showed that the media painted a picture that psychotic disorders and other mental conditions were not being taken seriously by the policymakers.

Another common frame in the news stories was on the incidence and prevalence of psychotic disorders. According to the DSM-5, the prevalence of schizophrenia spectrum disorders is 0.3–0.7% of the population with variations across regions and countries [3]. Many of the new stories cited medical experts while others cited Wikipedia. One news story citing Wikipedia indicated a prevalence of 0.6–1% while another news story referencing a physician cited the prevalence as 1% of the population. Yet another news story cited a rate of 2%. Although the rates cited by the news stories seemed to be slightly higher than that by the DSM-5, it is consistent with the documented evidence. In this case, the framing of the prevalence of schizophrenia did not appear to have exaggerated the true prevalence of the condition. Therefore, one could argue that the framing of the prevalence of schizophrenia in the news stories was close to accurate.

One of the problem frames in the news stories was the shortage of mental health professionals notably psychiatrists and psychologists. These stories focused on the low clinician to patient ratios across the country. The problem of shortage of health professionals was framed as pervasive and a symptom of larger problems within the health system. These problems included few mental health professionals being trained every year, few mental health care facilities and the problem of trained health care professionals preferring to work in the private health sector. This framing seemed to be consistent with the literature that suggested that there is an artificial shortage of mental health professionals as many qualified and registered mental professionals choose to work in private practice, leaving the majority of the South African population, who depend on the public health facilities, without access to basic mental healthcare services [26].

The dominant frame of the cause of schizophrenia and other psychotic disorders in the news stories was that it is mainly caused by the use of psychoactive drugs/substances, particularly Cannabis. Several news stories reported on new and on-going research that found a link between Cannabis and psychosis. Some news stories reported that people who have a family history of psychosis were more likely to develop psychosis if they smoked Cannabis. This framing is consistent with literature which suggests that the tetrahydrocannabinol component of cannabis could be a factor that triggers psychosis in at-risk populations [27, 28].

Still, it is interesting that most news stories noted that the exact causes of schizophrenia and psychosis were not well understood even in the science field, a position that is consistent with the literature [29]. Indeed, research has not identified one single cause of schizophrenia [1]. Studies have shown that the causes of schizophrenia are complex and multifactorial, with contributions from physical, genetic and environmental factors [30, 31]. Psychosocial factors may also affect the onset and course of schizophrenia [1].

It is notable that only few news stories discussed therapies for schizophrenia. This may limit health literacy and tend to mislead the public into having a limited understanding of the potential therapies available for the treatment of schizophrenia. Enhanced health literacy may likely encourage health seeking behaviour among people living with schizophrenia and their significant others.

With regards to moral judgements, many news stories called for an increase in the number of mental health professionals and the provision of more psychosocial and mental health services, specifically an increase in the capacity of mental health institutions and community mental health services. This framing seemed to be consistent with the literature that suggested that community mental health services were cost-effective in terms of improved population coverage [32]. Many of the news stories also called for the provision of services that would ensure early detection of mental health problems. With regards to suggested remedies, de-institutionalisation was a dominant frame in the news stories. However, some news stories opposed this policy by framing it as being primarily aimed at saving cost. Some news stories also framed de-institutionalization as not properly implemented because people living with schizophrenia spectrum and other psychotic disorders ended up being re-institutionalised.

A number of limitations of the study must be noted. First, we limited our study to the print media whereas other types of media such as radio, television and social media reporting issues relating to schizophrenia and other psychotic disorders were excluded. Second, we included only print media available on the SA media database on SABINET whereas there might be print media not available on this database. Third, we included new stories published in English but did not include those published in local South African languages. Print media published in local South African languages may be important sources of information for a wide variety of people in South Africa. Fourth, we focused on news stories published over an 11-year period immediately following the enactment of two major mental health policies. We note that more current media reporting may differ from media reports from the period that we analysed.

## Conclusion

The two Mental Health policies have clear provisions that seek to, among other goals, ensure that appropriate care, treatment and rehabilitation are provided to people living with mental health conditions. The policies also emphasize that individuals with mental health conditions should not be discriminated against, stigmatized or abused. However, the media frames on problems and causes contained misconceptions about schizophrenia which may potentially perpetuate stigmatisation, discrimination, and social rejection towards individuals with lived experiences of schizophrenia. The findings suggests that there are still challenges that exist post the implementation of the Mental Health policies including shortage of mental health professionals and lack of funding for mental health initiatives. Our findings highlighted the need for collaboration between researchers and the media to enhance opportunities for improved and more nuanced reporting of mental health issues.

## Data Availability

The datasets generated and/or analysed during the current study are available in the SA Media – located on the SABINET search engine.
